# Increased medial tibial slope is a possible risk factor for patellar cartilage lesions

**DOI:** 10.1002/jeo2.70058

**Published:** 2024-11-27

**Authors:** Kai Hoffeld, Jan P. Hockmann, Christopher Wahlers, Peer Eysel, Johannes Oppermann

**Affiliations:** ^1^ Department of Orthopaedic, Trauma and Plastic Surgery, Faculty of Medicine and University Hospital of Cologne University of Cologne Cologne Germany

**Keywords:** axial deviation, cartilage damage, cartilage lesions, cartilage repair, knee axis, risk factor, sagittal plane, tibial slope

## Abstract

**Introduction:**

Axial malalignment in the coronal plane has been identified as a significant risk factor for knee cartilage damage, leading to osteoarthritis progression. However, the impact of sagittal axial deviation on cartilage damage remains underexplored. Biomechanical studies have suggested that an increased tibial slope leads to altered pressure distribution in the articular cartilage, potentially contributing to cartilage damage. Despite these biomechanical insights, clinical evidence linking increased tibial slope to cartilage damage is lacking.

**Methods:**

This retrospective study focuses on patients who underwent surgical cartilage transplantation between January 2016 and July 2023. A total of 108 patients were divided into two groups based on the presence or absence of other pathologies contributing to cartilage damage. Clinical data, including tibial slope measurements from lateral radiographs, were collected. A further subgroup‐matched pair analysis was conducted comparing cases with patellar lesions and healthy knees. Statistical analysis compared tibial slope values between groups and assessed correlations between tibial slope and cartilage lesion grade.

**Results:**

Patients without other identifiable pathologies exhibited a significantly higher medial tibial slope compared to those with known causative factors for cartilage damage (*p* < 0.05). Cartilage damage, particularly in the patellar region, was more prevalent in patients with an increased tibial slope. Patients with patellar lesions had a significant increased slope than healthy controls (*p* < 0.05). However, there was no significant correlation between cartilage lesion grade and tibial slope.

**Conclusion:**

The study identified increased medial tibial slope as a possible independent risk factor for cartilage damage in the knee, especially in the patellar region.

**Level of Evidence:**

Level IV.

**Abbreviations**
ACL: anterior cruciate ligament
ACT: autologous chondrocyte transplantation
BMI: body mass index
HTO: high tibial osteotomy
ICC: intraclass correlation
LFC: lateral femoral condyle
LTC: lateral tibial condyle
MFC: medial femoral condyle
MTC: medial tibial condyle
PFJ: patellofemoral Joint
SG1: subgroup 1
SG2: subgroup 2
TTTG: Tuberositas‐Tibiae‐Trochlea‐Groove‐Index

## INTRODUCTION

Axial malalignment in the coronal plane is a significant risk factor for cartilage damage in the knee [20]. In contrast, the effect of sagittal axial deviation on the development and progression of cartilage damage has not yet been sufficiently analysed. Agneskirchner et al. were the first who described how flexion osteotomy of the proximal tibia, combined with an increase in the posterior tibial slope, affects pressure distribution within the articular cartilage of the knee [1]. In their cadaveric study, they applied axial force after gradually increasing the posterior tibial slope of knee specimens by an anterior flexion osteotomy of the proximal tibia. This resulted in a decreased load on the posterior and an increased load on the anterior half of the medial tibial plateau [1]. These findings were further supported by another cadaveric study by Black et al. [4]. In a similar biomechanical setting with cadaveric knees, the authors investigated the changes in joint kinematics after an anterior flexion osteotomy of the proximal tibia and a gradually increased tibial slope. They suggested that an increased tibial slope results in altered tibiofemoral and patellofemoral kinematics, leading to the centre of pressure shifting toward the anterior part of the joint. This anterior shift in the centre of pressure affects the distribution of forces within the knee, potentially impacting cartilage pressure and joint stability. This raised the question of whether the findings from the preliminary biomechanical studies could be transferred to clinical observations.

The literature research revealed only one clinical study which investigated the relationship between an increased tibial slope and the development of cartilage damage [22]. Khan et al. found that an increased lateral tibial slope correlated with cartilage damage to the medial patella facet and the lateral tibial plateau. The medial tibial slope was not analysed in their study. The relationship between increased medial tibial slope and the development of cartilage damage has, therefore, not yet been adequately described.

The aim of this study is to analyse a possible correlation between increased medial tibial slope and the specific localisation of cartilage damage in order to identify increased tibial slope as a potential risk factor for cartilage damage. Based on the findings of existing literature, a correlation between an increased medial tibial slope and cartilage damage in the patellar region was expected. However, as previously mentioned, there is no clinical evidence addressing this specific question. Therefore, this study investigates whether increased medial tibial slope is associated with localised cartilage damage, especially in the patellar area.

## METHODS

The present study is a retrospective analysis of two cohorts. The level of evidence is level IV. Ethical approval for this study was given by the Institutional Review Board of the University of Cologne (ID‐number: ID‐number: 24‐287533‐retro).

All patients who underwent surgical cartilage transplantation autologous chondrocyte transplantation (ACT) and minced cartilage (applied regions: patellar, trochlear, medial or lateral femur condyle) between 1 January 2016 and 31 July 2023 were selected from the digital database of the Department of Orthopaedic and Trauma Surgery at the University Hospital of Cologne. Relevant clinical data were collected from the study collective. In addition to epidemiological data such as age at surgery, gender and body mass index (BMI), relevant previous damage and degenerative changes in the affected knee as well as the exact localisation of the cartilage damage were taken into account. The latter could be precisely determined based on the arthroscopic findings documented in the operative report. Two study groups (Group A and Group B) were formed based on the recorded clinical data. Group A was formed from patients in whom no previous damage (ligamentous injury [12], meniscal injury [25], axial deviation in the coronal plane of more than 3° from the normal value of the medial proximal tibial angle [85–90°] and lateral distal femur angle [85–90°] [20]), fractures of the distal femur, patella and proximal tibia [11], previous operations, Ahlbäck's disease [27], osteochondrosis dissecans [19] or traumatic cartilage damage was found and, therefore, no causal pathology could be identified as the cause of the cartilage damage present. Group B was formed from all patients in whom at least one of the above‐mentioned pathologies was known. This should ensure that all confounders were excluded when the tibial slope was analysed as a possible risk factor for cartilage damage.

For our data analysis, 186 patients who initially received a cartilage transplant were identified. A total of 108 patients were included in the data analysis after applying the exclusion criteria, of whom 57 patients were assigned to Group A and 51 patients were assigned to Group B.

**Figure 1:**
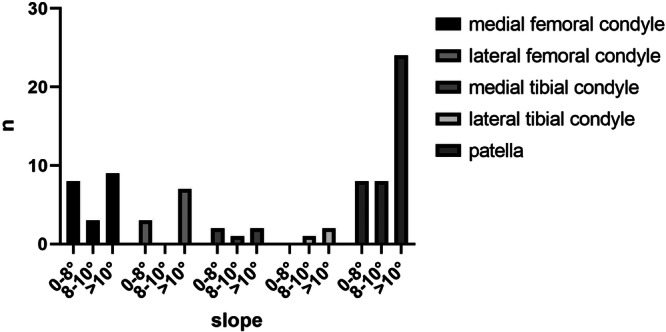
Number of cartilage lesions in specific regions for different slope levels in Group A.

The distribution pattern of cartilage lesions was reported for both groups. Since Group B represented a very heterogeneous study group and the respective pathologies led to cartilage damage at different localisations, a further assessment of the distribution of the lesions' localisation was checked regarding the altitude of the tibial slope only for Group A. Therefore, the lesion occurrence was grouped for 0–8°, 8–10° and >10° of slope (see Figure 1). The ranges were chosen based on the average values for the slope of the affected group and the healthy group of the following subgroup (SG) analysis.

For the SG analysis, two subgroups were formed. SG1 consisted of all cases of Group A with cartilage damage at the patella. SG2 consisted of patients with healthy knees. SG2 was formed as matched pairs of SG1 in terms of age and sex. In order to gather the necessary 42 patients for the matched pair analysis, the patients were derived from the emergency room's patient pool. Patients who underwent a radiological examination of the knees due to a knee contusion were further screened for pre‐existing conditions of the corresponding knee, according to the exclusion criteria mentioned above. If any of these conditions were present, the patient was excluded as a candidate for the matched pair analysis and replaced by another. After applying the clinical exclusion criteria, the radiologic exclusion criteria (see below) were applied as well. The matching was done by sex and age, matching the year of birth in all cases. For SG1, the grade of the cartilage lesion based on the arthroscopic findings documented in the operative report was provided. In the reports, the International Cartilage Research Society (ICRS) classification was applied to determine the grade of the cartilage lesion and was documented accordingly [5]. The axial alignment of the coronal plane was examined on the available radiographs (i.e., whole‐leg radiograph and standard knee radiograph in two planes). This ensured that no patients with a pathological alignment of the coronal axis were included in any of the study groups.

The medial tibial slope was measured for all study groups.

The patella height was determined by calculating the Insall‐Salvati ratio in all lateral radiographs of SG1 and the matched pair group. An overview of the epidemiological data can be found in Table 1.

**Table 1:** Epidemiological data Groups A and B.

G	Group A	Group B	Difference A/B
Male/female	24/33	21/30	
Age (years)	35	29	6 (*p* < 0.05)
BMI (kg/m^2^)	27	28	1 (*p* > 0.05)
Slope (degree)	10.6	8.8	1.8 (*p* < 0.05)

Abbreviation: BMI, body mass index.

### Radiographic assessment

The tibial slope is defined as the angle formed by the intersection of the posterior slope of the tibial plateau and a line perpendicular to the diaphyseal shaft axis of the tibia. The posterior tibial slope was determined from the lateral radiograph of the knee joint using the two‐circle method [8].

**Figure 2:**
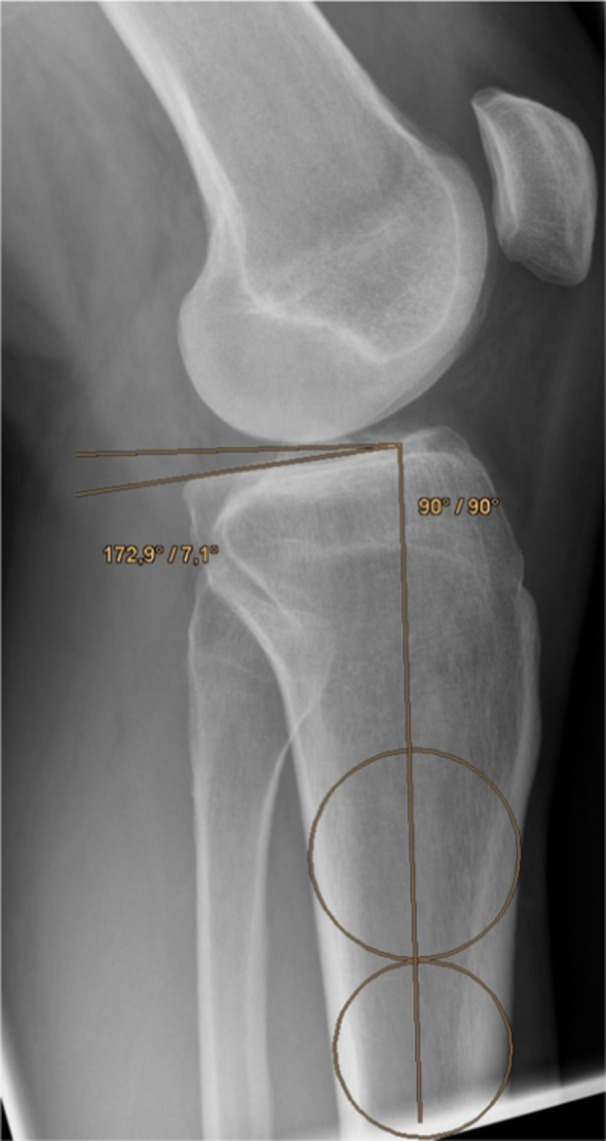
Radiographic assessment on lateral knee radiograph.

The diaphyseal shaft axis was determined by drawing a vertical line through the centres of two circles in the tibial diaphysis, one circle below the tibial tuberosity and the second circle as far distally as possible. The angle between the tangent applied to the respective medial tibial plateau and the perpendicular to the defined diaphyseal axis was then determined, which ultimately measured the medial tibial slope (Figure 2).

Since malrotation of the knee in the radiograph leads to additional measurement errors of the tibial slope, the intercondylar distance should be <10 mm [21]. Therefore, the posterior intercondylar distance was used to determine the extent of radiographic malrotation.

The length of the diaphyseal axis depicted on the radiograph should be at least 125 mm long to avoid measurement errors [14], which is why this was also recorded in our study.

**Figure 3:**
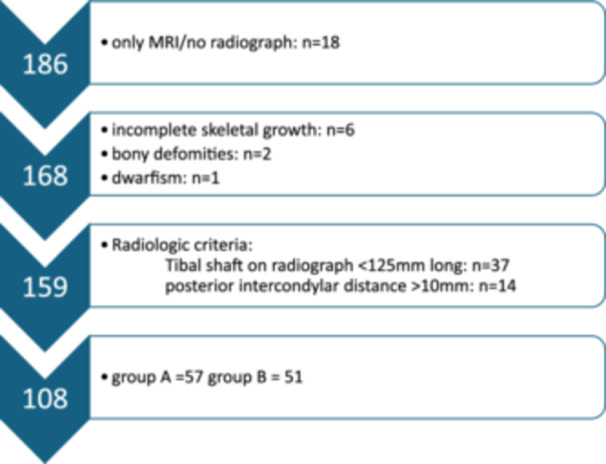
Flowchart of patient inclusion/exclusion.

If the available radiographs did not meet the above‐mentioned quality criteria required for a valid determination of the tibial slope, the patients were excluded from the study. Patients for whom only an MRI and no radiographs were stored in the system were also excluded. Further exclusion criteria were incomplete skeletal growth, short stature and other bony deformities such as exostoses, which would influence the measurement. The inclusion and exclusion process based on the radiologic parameters is shown in Figure 3.

### Data analysis

A power analysis was conducted for the difference between two dependent means (matched pairs) with the software G*Power 3.1.9.7. The power was calculated with an estimated effect size of *d* = 0.8, which represents a medium effect size (cohen's *d*: 0.2–0.5 = low effect size, 0.5–0.8 = medium effect size, >8 = large effect size), an *α*‐error of 0.05 and a power level of 0.95. In this way, the sample size was calculated as 19 patients per group, the actual calculated power was 0.96.

The radiographs were each measured twice by two independent observers (6th‐year resident of orthopaedic surgery and orthopaedic consultant) using IMPAX EE R20XIX SU1 according to the two‐circle method, and then both the interrater correlation coefficient and the intrarater correlation coefficient were calculated. The interrater agreement between the two observers as well as the intrarater agreement for both observers for the slope measurement was calculated using the intraclass correlation (ICC) according to Shrout and Fleiss [30] (absolute agreement). ICC benchmarks were used as proposed by Cicchetti [6] (poor: ICC < 0.4; moderate: 0.4–0.59; good: 0.6–0.74; excellent: 0.75–1.0).

Measurement accuracy was calculated by standard error of measurement in degrees. The standard error of means (SEM) was calculated based on the formula: $\mathrm{SEM}=\mathrm{SD}\times \sqrt{1-\mathrm{ICC}}$.

Statistical analysis was performed with IBM SPSS Statistics Version 29.0 for Mac (IBM Corp.).

We report median (range) and/or mean ± standard deviation. A value of *p* < 0.05 was considered to be statistically significant. For regression and correlation evaluation, statistical outliers were identified and eliminated.

Correlation between the grade of the cartilage defect and the tibial slope was analysed by Spearman's rank correlation coefficient. All other correlations were analysed by the Pearson correlation coefficient. Besides *n* > 30 being widely accepted for assuming normal distribution, we also calculated Kolmogorov–Smirnov and Shapiro–Wilk with indication of a normal distribution for the slope.

Graphics were done with GraphPad Prism 9.5.1.

## RESULTS

In Group A, the average medial posterior tibial slope was 10.9°. In Group B, the average medial posterior tibial slope was 8.8°. A more detailed analysis of the cartilage damage revealed the following results:

In Group A, which consisted of patients without any preexisting pathologies of the knee, there were 20 cases with defects on the medial femoral condyle (MFC), 10 cases on the lateral femoral condyle (LFC), five cases on the medial tibial condyle (MTC) and three cases on the lateral tibial condyle (LTC). Additionally, there were 40 cases with defects at the patella in Group A.

**Figure 4:**
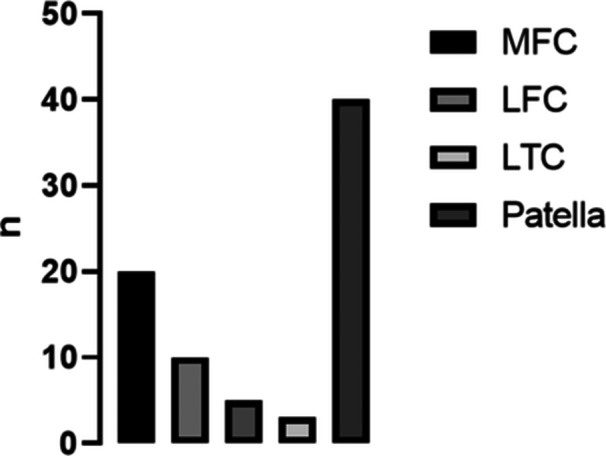
Number of cartilage lesions for specific location in Group A. LFC, lateral femur condyle; LTC, lateral tibial condyle; MFC, medial femur condyle; MTC, medial tibial condyle.

In Group B, which consisted of patients with at least one of the above‐mentioned specific preexisting pathologies, there were 19 cases with defects on the MFC, four cases on the LFC, four cases on the MTC and three cases on the LTC. Furthermore, there were 26 cases with defects at the patella in Group B. Figure 4 shows the distribution of cartilage damage of Group A. There was no correlation between age (*p* > 0.05) or BMI (*p* > 0.05) and observed slope.

The difference between the two mean values of the tibial slope of Groups A and B was 1.8° at *p* < 0.05. The difference of 1.8° can, therefore, be regarded as significant.

The difference in mean age was 6.44 years at *p* < 0.05. Thus, the difference in age can be considered statistically significant.

The difference in BMI was 0.88 kg/m^2^ at *p* > 0.05. The difference in BMI is, therefore, not significant.

The SGs of patients with cartilage lesions at the patella (SG1) and the control group of healthy knees (SG2) consist of 32% (13) male and 68% (27) female individuals. The average medial posterior tibial slope was 10.9° for SG1 and 7.3° for SG2. The matched pair analysis revealed an average difference of the tibial slope between the matched pairs of 3.6 ± 4.3° (*p *< 0.05), see Table 2. In eight patients of SG1, the tibial slope was 0–8°, 8 patients had 8–10° and in 24 cases, the slope was >10°. The grade of the cartilage damage, classified by ICRS classification, revealed zero grade I, four grade II, 19 grade III and 13 grade IV lesions at the patella. Four cases were without classification. There was no correlation between grade of the cartilage defect and an increased tibial slope (*p* > 0.05).

**Table 2:** Average slope and slope difference for matched pair analysis.

	Patella defect	Matched pairs	Difference
Male/female	13/27	13/27	
Insall‐Salvati	0.92	0.89	0.03 (*p* > 0.05)
Slope (degree)	10.9	7.3	3.6 (*p* < 0.05)

The patella cases were checked for involvement of the trochlea groove. In 10 cases, a cartilage lesion was found in the area of the trochlea, with four cases showing only slight signs of abrasion and six cases showing manifest cartilage damage. Of the six cases of actual cartilage damage, three cases were ICRS grade I, one case was ICRS grade II, one case was ICRS grade III and one case was ICRS grade IV. In 30 cases, there were unremarkable cartilage findings. There was no correlation between the tibial slope and the presence of cartilage damage in the trochlear groove.

The intrarater correlation coefficient, that is, the comparison between the first and second measurements of one observer in each case, was 0.81 for the first observer and 0.73 for the second observer, which is in the excellent and good range. The interrater correlation coefficient, that is, the comparison between the measurements of both observers, was 0.78, which is also in the excellent range.

The SEM for the measurement of the slope for the first rater was 0.64° and for the second rater was 0.84°. For the interrater correlation, it was 0.76°

The average Insall‐Salvati ratio was 0.92 in SG1 and 0.89 in the matched pairs. The difference of the Insall‐Salvati ratio between both groups was 0.03 (95% confidence interval −0.04 to 0.1), *p* > 0.05.

## DISCUSSION

The study investigated the relationship between increased medial tibial slope and cartilage damage in the knee, particularly focusing on the patellar region. The findings revealed a significant association between an increased tibial slope and cartilage damage, particularly in cases where no other identifiable pathology could explain the damage. This suggests that the sagittal plane, specifically the tibial slope, may play a crucial role in the development of cartilage lesions, alongside the well‐established risk factors in the coronal plane.

The sagittal plane has not been given special consideration in the development of cartilage damage of the knee, yet. An increased medial and lateral slope has been identified as a major risk factor for ruptures of the anterior cruciate ligament (ACL) [7, 9, 18, 28]. Studies of knee kinematics have shown that an increased tibial slope increases anterior translation of the tibia and increases the force exerted on the ACL [1, 8, 13, 15, 29] and furthermore decompresses the posterior joint compartment, reduces posterior translation of the tibia and reduces the force exerted on the posterior cruciate ligament [1, 16, 28]. Considering the significant impact of an altered tibial slope on knee joint kinematics, it is likely that increased translational movements lead to heightened cartilage stress due to changes in pressure distribution and force magnitude. This is supported by findings that changes in contact location and relative velocity between the femoral and tibial cartilage at the joint surface are associated with cartilage degeneration [2, 3]. The literature reports an average tibial slope of 8.2° (±2.8°) −8.6° (±2.6°) in healthy subjects [18, 28]. In our study, the average slope in Group B was 8.8°, which is roughly in the range of the physiological slope. In addition, the average tibial slope of the control group of healthy knees (SG2) was 7.3° (±1.5°). In contrast, the slope in Group A, that is, in the group in which no pathology could be identified that could have caused the cartilage damage, was 10.6° and thus significantly increased. When looking at the patella cases (SG1) the average slope was even higher with 10.9°. Based on the existing evidence from biomechanical studies [1, 4], as described in the introduction, a correlation between an increased slope and cartilage damage in the patellar region was expected. The assumption was reaffirmed when the cartilage lesion cluster was examined. A significant number of lesions were found in the patella region, especially for a tibial slope of 10° or more. This is in line with the findings by Khan et al. [22], who found that an increased lateral tibial slope shows a statistically significant association with worsening cartilage degenerative changes in the patella. The increased occurrence of cartilage lesions at the patellar site may be explained by an increased contact pressure of the patellofemoral joint (PFJ). Studies have shown that in medial open wedge high tibial osteotomy (HTO), the tibial slope is increased [26, 33] and results in greater patellofemoral contact pressure [32]. Other studies have shown that the patellofemoral kinematics are also changed after increasing the tibial slope in HTO by increasing the medial patella tilt [4, 10]. These effects would explain the relation between the occurrence of patellofemoral cartilage damage and an increased tibial slope. In contrast, another study investigating the effect of HTOs on the PFJ showed that the PFJ is relieved after HTO if the slope remains unchanged [31]. The average tibial slope in the study was 10–11° and changed in average 0.3° postoperatively. This contradicts the results found in our study, where we found a significant accumulation of cartilage damage in the PFJ at 10° of slope or more. However, it should be noted that the changed anatomical alignment of the HTO changes several biomechanically relevant axes and angles. The study cites the change in the Tuberositas‐Tibiae‐Trochlea‐Groove‐Index (TTTG) distance and the Q angle (angle between the quadriceps muscle and the patellar tendon) as the most statistically relevant factors [31]. It is, therefore, questionable whether the off‐loading effects on the PFJ of the altered TTTG distance and Q angle overlap the suggested straining effects on the PFJ of the slope of more than 10°. Consideration of the isolated effect of the tibial slope remains difficult when summarising the findings from the literature and indicates the need for further studies to investigate the biomechanical effects of an altered slope.

When looking for pathologic alterations in patella height in the study groups, no abnormalities were detected, as both Insall‐Salvati ratios were in the physiologic range. BMI showed no statistically significant difference in both groups. Hence, patella alta or baja as well as BMI could be excluded as confounders. In addition to these excluded confounders, the literature also describes other factors that can cause patellar cartilage damage. Murakami showed that postoperative quadriceps weakness is a risk factor for patellofemoral articular cartilage lesions after ACL reconstruction [24], which is in line with other studies describing that quadriceps muscle strength affects articular cartilage injury of the PFJ [17, 23]. Due to the retrospective nature of the study, it was impossible to analyse this effect in our cohort. An influence of the force of the extensor apparatus on the patellar cartilage lesions can, therefore, neither be proven nor disproven in our study but should be considered in patients with complaints in the PFJ. Another risk factor for patellar cartilage damage found by Murakami is the male sex [24]. Our cohort with cartilage lesions of the patellar consists of 16 men compared to 26 women. A relevant influence on our data can, therefore, be neglected.

Group A showed a significant greater average age. In this context, it is conceivable that the cartilage damage caused by an increased slope, in contrast to other causative pathologies, only becomes apparent later in life and thus explains the higher average age in Group A. However, there is insufficient evidence to support this hypothesis.

Overall, the presented study has some limitations: First, the retrospective design of the study may have been associated with confounding factors that led to the statistically significant differences. For example, a selection bias could have caused the statistically significant age difference or the pronounced difference in slope between Group A and the matched pairs. Other biases that cannot be ruled out could be a prior trauma of the knee not reported in patients' medical history and a recall bias of prior issues. Second, the number of data sets that were included in the analysis after applying all exclusion criteria was not large enough to statistically verify the tested hypothesis. The results and conclusion give trends for evidence but more studies with a larger cohort must prove, what we have found.

## CONCLUSIONS

The study identified increased medial tibial slope as a possible independent risk factor for cartilage damage in the knee, especially in the patellar region.

## AUTHOR CONTRIBUTIONS

**Kai Hoffeld**: Conceived and designed the analysis; collected the data; wrote the paper. **Jan P. Hockmann**: Contributed data and analysis tools; performed the analysis; editing. **Christopher Wahlers**: Collected the data. **Peer Eysel**: Supervision; consultation. **Johannes Oppermann**: Supervision; editing; consultation.

## CONFLICT OF INTEREST STATEMENT

The authors declare no conflict of interest.

## ETHICS STATEMENT

Ethical approval for this study was given by the Institutional Review Board of the University Cologne (ID‐number: 24‐287533‐retro). Due to retrospective character of this study, no informed consent was needed.

## Data Availability

The data that support the findings of this study are available from the corresponding author upon reasonable request.
